# Analysis of adenovirus trans-complementation-mediated gene expression controlled by melanoma-specific TETP promoter *in vitro*

**DOI:** 10.1186/1743-422X-7-175

**Published:** 2010-07-29

**Authors:** Alessandra Curioni Fontecedro, Verena Lutschg, Ossia Eichhoff, Reinhard Dummer, Urs F Greber, Silvio Hemmi

**Affiliations:** 1 Faculty of Mathematics and Natural Sciences, Institute of Molecular Life Sciences, University of Zürich, Winterthurerstrasse 190, CH-8057 Zürich, Switzerland; 2 Faculty of Mathematics and Natural Sciences, Institute of Molecular Life Sciences, Zürich PhD Program in Molecular Life Sciences, University of Zürich, Winterthurerstrasse 190, CH-8057 Zürich, Switzerland; 3 Department of Dermatology, University Hospital of Zürich, Gloriastrasse 31, CH-8091 Zürich, Switzerland; 4 Faculty of Mathematics and Natural Sciences, Institute of Molecular Cancer Research, Cancer Biology PhD Program, University of Zürich, Winterthurerstrasse 190, CH-8057 Zürich, Switzerland

## Abstract

**Background:**

Human adenoviruses (Ads) have substantial potential for clinical applications in cancer patients. Conditionally replicating adenoviruses (CRAds) include oncolytic adenoviruses in which expression of the immediate early viral transactivator protein E1A is controlled by a cancer cell-selective promoter. To enhance efficacy, CRAds are further armed to contain therapeutic genes. Due to size constraints of the capsid geometry, the capacity for packaging transgenes into Ads is, however, limited. To overcome this limitation, the employment of E1A-deleted replication-deficient viruses carrying therapeutic genes in combination with replication-competent CRAd vectors expressing E1A *in trans *has been proposed. Most trans-complementing studies involved transgene expressions from strong ubiquitous promoters, and thereby relied entirely on the cancer cell specificity of the CRAd vector.

**Results:**

Here we tested the trans-complementation of a CRAd and a replication-deficient transgene vector containing the same cancer cell-selective promoter. Hereto, we generated two new vectors expressing IL-2 and CD40L from a bicistronic expression cassette under the control of the melanoma/melanocyte-specific tyrosinase enhancer tyrosinase promoter (TETP), which we previously described for the melanoma-specific CRAd vector AdΔEP-TETP. These vectors gave rise to tightly controlled melanoma-specific transgene expression levels, which were only 5 to 40-fold lower than those from vectors controlled by the nonselective CMV promoter. Reporter analyses using Ad-CMV-eGFP in combination with AdΔEP-TETP revealed a high level of trans-complementation in melanoma cells (up to about 30-fold), but not in non-melanoma cells, unlike the AdCMV-eGFP/wtAd5 binary vector system, which was equally efficient in melanoma and non-melanoma cells. Similar findings were obtained when replacing the transgene vector AdCMV-eGFP with AdCMV-IL-2 or AdCMV-CD40L. However, the combination of the novel AdTETP-CD40L/IL-2 vector with AdΔEP-TETP or wtAd5 gave reproducible moderate 3-fold enhancements of IL-2 by trans-complementation only.

**Conclusions:**

The cancer cell-selective TETP tested here did not give the expected enforceable transgene expression typically achieved in the Ad trans-complementing system. Reasons for this could include virus-mediated down regulation of limiting transcription factors, and/or competition for such factors by different promoters. Whether this finding is unique to the particular promoter system tested here, or also occurs with other promoters warrants further investigations.

## Introduction

Cancer immunotherapy is an experimental approach for treatment of cancer patients. It aims at evoking immune-based responses against malignant cells by activating and recruiting cells from the innate and adaptive immune system, including T cells that recognize tumor-specific antigens [1]. Virus-mediated gene transfer has been widely used to enhance the susceptibility of cancer cells to immunotherapy. Therapeutic genes expressed by viral vectors included a broad number of immune modulators, such as e.g. granulocyte macrophage colony stimulating factor (GM-CSF), interleukin-2 (IL-2), interferons or CD40 ligand (CD40L) [2-4]. These approaches have proven to be inefficient, however, since most tumors express weak tumor antigens and also lack co-stimulatory molecules necessary for induction of cellular immunity, and evade immune recognition. An additional major limitation of cancer immunotherapy has been the low rates of gene transfer.

Strategies to improve both, the potency of immune recognition of cancer cells and the efficacy of gene therapy are clearly required to successfully employ the promising concept of cancer immunotherapy. One way to enhance the duration of therapeutic gene expression is to increase viral spreading [5], for example by replacing non-replicating therapeutic virus vectors with armed oncolytic viruses, which replicate selectively within cancers and also express therapeutic genes [6-8]. Therapeutic genes include prodrug converting enzymes, suicide genes, and immunostimulatory proteins. The most widely used oncolytic viral vectors have been derived from non-integrating parental viruses, such as vaccinia virus, herpes simplex virus, measles virus and human Ad [9]. Human Ads have an excellent safety profile in cancer gene therapy [10]. In addition, they are easy to produce in large amounts, and efficient infection is possible with vectors derived from various serotypes or by tropism engineering [11]. The latter is based on the availability of adequate methods to generate recombinant vectors of choice, including CRAds for cancer treatment [12,13].

The number of inserted therapeutic genes is, however, limited for Ad due to the packaging capacity of the viral capsid [14]. One promising way to overcome this limitation is to use trans-complementing co-replication, where a CRAd is mixed with a second, E1-deleted and therefore replication-deficient (RD) vector expressing the therapeutic gene(s) of interest. In such a system, the E1A gene expressed by the first vector complements the second vector *in trans*, which gives rise to efficient replication of both vectors, thereby strongly increasing the DNA copy number on a per cell basis. This concept was first confirmed by combining transduction of an E1-deleted Ad with transfection of a plasmid containing the E1 genes and subsequent production of progeny virus and enhanced viral transgene expression [15,16].

Subsequently, numerous variations of the binary virus system have been tested. The group of Alemany was the first to combine two defective viruses, a RD E1-deleted virus and a helper-dependent virus containing the E1 genes under the control of the liver tissue-specific promoter and demonstrated oncolytic spread following injection of a 1:1 mix into human hepatocarcinoma mouse xenograft model [17]. Several groups used RD Ad vectors expressing reporter genes such as β-galactosidase, luciferase or eGFP to show enhancement of virus replication and cell spreading [18-23]. More recently, this system has led to new exciting applications for non-invasive *in vivo *imaging of tumor spread and assessment of Ad replication [24-27]. Therapeutic genes utilized in binary vector systems included prodrug converting enzymes such as herpes simplex virus-thymidine kinase [28,29] and P450 enzyme [21], suicide genes like Bcl-xs [30], p53 [31,32], p27 [25], tumor necrosis factor α-related apoptosis-inducing ligand [23,33-36], dominant-negative insulin-like growth factor-1R [22], antiangiogenic soluble vascular endothelial growth factor receptor 2-Fc [26,37], and immunostimulatory proteins like GM-CSF [33,38], IL-2 and IL-12 [39]. In most studies, co-administration of RD therapeutic vectors and CRAds was also tested in *in vivo *xenotransplant models. Improved oncolytic efficacy was found and in some cases lead to complete and long lasting regression, which was not achieved when using the individual viral vectors alone [21-26,28,29,33-37,39].

The currently available dual vector co-replication systems control the expression of the therapeutic genes by strong ubiquitous promoters lacking tissue or tumor specificity. In this study we suggested to combine a CRAd and a RD vector containing the same cancer cell-selective promoter, and to test, whether a strong enhancement of transgene expression can be achieved by trans-complementation. The results presented here show that when using the cancer cell-selective TETP promoter previously described for our melanoma/melanocyte-specific CRAd vector [40] in combination with two novel RD vectors expressing IL-2 and CD40L from a bicistronic expression cassette, only moderate 3-fold enhancements of IL-2 or CD40L were obtained, whereas controls including the CMV promoter allowed much stronger expression enhancement in the Ad vector co-replication system. Possible reasons for this finding are discussed.

## Materials and methods

### Cells

The primary melanoma short time culture cells M000301 were grown in RPMI 1640 plus 8% FCS [41]. All other cell lines including the human embryonic retina cell line 911, the human lung carcinoma cell line A549, the human cervical carcinoma cell line HeLa and the human colon carcinoma cell lines SW480, DLD-1, the human melanoma cell lines M21-L4, MeWo and SK-Mel23 were grown in DMEM plus 8% FCS [40,42,43]. All cell lines were routinely screened for the absence of mycoplasma contamination.

### Viruses

The recombinant, first-generation, E1/E3-deleted Ad5-based vectors AdTETP-IL-2/CD40L and AdTETP-CD40L/IL-2 expressing mouse IL-2 and CD40L were generated as described previously [42]. Briefly, homologous recombination was performed in 911 cells between a transfer plasmid containing the transgene under the control of TETP and a genomic *Cla *I DNA fragment isolated from AdMLP-*lacZ*. To generate the transfer plasmids, a *Kpn *I -*Bgl *II (blunt) TETP fragment of 1197 bp was released from the pGL3-4xTETP plasmid [40] and was cloned in a first step into the *Kpn *I -*Bam *HI (blunt)-restricted transfer plasmid pAdCMVΔlacZ-lnk1 to replace the CMV promoter. The resulting pAdTETPΔlacZ-lnk1 carried an E1 deletion from bp 449 to 3323. For the generation of the two bicistronic expression cassettes, the IL-2 and CD40 ligand-encoding fragments were linked by an internal ribosomal entry site (IRES) derived from encephalomyocarditis virus [44]. For this, the CD40L sequence was PCR-amplified using the forward primer 5'-GCGCATGCGGTCTCCCATGATAGAAACATACAGCCAAC-3' and the reverse primer 5'-GCGCCTCGAGTGCAGCCTAGGACAGCGCACTG-3', introducing a terminal *Bsa *I site with a *Nco *I overhang-matching sequence at the 5'-end and a terminal *Xho *I site at the 3'-end. The *Bsa *I - *Xho *I fragment was cloned between the *Nco *I - *Xho *I-digested pBlusecript-IRES [44]. In a second step, a *Pst *I (blunt) - *Spe *I (blunt) IL-2 sequence containing fragment was cloned into the *Xba *I-digested (blunt) pBlusecript-IRES-CD40L. For the generation of the inverse construct, the IL-2 sequence was first PCR-amplified using the forward primer 5'-GCGCATGCGGTCTCGCATGTACAGCATGCAGCTCG-3' and the reverse primer 5'-GCGCCTCGAGGAGCCTTATGTGTTGTAAGCAG-3', followed by the same cloning strategy as above. A *Pst *I (blunt) - *Sal *I (blunt) CD40L sequence containing fragment was cloned into the *Xba *I-digested (blunt) pBlusecript-IRES-IL-2. For the final ligation, the *Not *I (blunt) - *Spe *I fragments of IL-2-IRES-CD40L and CD40L-IRES-IL-2 were cloned into the *Asc *I (blunt) - *Nhe *I- digested pAd-TETP.

Recombinant Ads were once plaque-purified, amplified and CsCl-purified. Viral titers were determined by plaque assay using 911 cells. Biological titers varied between 3 × 10^9 ^and 3 × 10^10 ^plaque forming units (pfu)/ml, when determined in a standard assay using 2 ml of medium and the cell layers contained in 6-well plates. For AdΔEP-TETP, AdCMV-lacZ, AdCMV-IL-2, AdCMV-CD40L, AdCMV-eGFP see references in Table 1.

**Table 1:** Adenoviruses used in this study

Virus name	E1 region	Titer (pfu/ml)	Reference
Wt Ad5	WT	1 × 10^11^	[40]

AdΔEP-TETP	E1A promoter deleted and replaced with TETP	1.4 × 10^10^	[40]

AdCMV-lacZ	deleted		[42]

AdCMV-IL-2	deleted	1.8 × 10^10^	[44]

AdCMV-CD40L	deleted	1.0 × 10^10^	[44]

AdCMV-eGFP	deleted	2.6 × 10^9^	[43]

AdTETP-IL-2/CD40L	TETP inserted downstream of E1A promoter	6.0 × 10^9^	This study

AdTETP-CD40L/IL-2	TETP inserted downstream of E1A promoter	5.2 × 10^9^	This study

### Expression analyses

For mouse CD40L and IL-2 expression analysis, triplicates of 1 × 10^5 ^cells seeded in 12-well plates were infected at multiplicities of infection (MOIs) ranging from 10 to 810. Medium was replaced 5 h post infection (p.i.) and cells were analyzed two days p.i.. Levels of CD40L were determined by flow cytometric analysis as described earlier [42] using R-PE-labeled anti-mouse CD40L (CD154) (09025B) and appropriate isotype controls (Pharmingen, San Diego, USA). FACS measurements consisted of 10000 viable cells per sample. Mouse IL-2 levels were determined in duplicates by ELISA (Mouse IL-2 kit, PIERCE ENDOGEN) using pooled samples.

For RT-PCR of Microphthalmia-associated bHLH-LZ transcription factor (Mitf), total RNA was extracted using TRIzol reagent according to manufacturer instructions (Invitrogen, Carlsbad, CA, USA). One microgram total RNA was used for cDNA synthesis using Promega's Reverse Transcription System with supplied poly d(T) primers according to manufacturer instructions (Promega, Madison, WI, USA). PCR was performed on 1 μg template cDNA using Roche's LightCycler DNA Master SYBR Green kit (Roche, Basel, Switzerland). Primers for 18sRNA were 5'-AAACGGCTACCACATCCAAG-3' and 5'-CCTCCAATGGATCCTCGTTA -3'. Primers for Mitf were purchased from Qiagen, QT00037737 (Qiagen, Hombrechtikon, Switzerland).

### Co-replication experiments

Experiments to assess co-replication potency on transgene expression were performed in 12-well plates using triplicate inputs. Four hours after seeding of 10^5 ^cells/well, serial 3-fold dilutions of GFP- or transgene-expressing viruses were added, followed immediately by addition of varying amounts of replication-competent viruses. Medium was replaced 5 h p.i. and cells were analyzed two days p.i. by flow cytometric analysis for GFP or CD40L, and by ELISA for IL-2.

## Results

### Novel Ad vectors containing TETP allow melanoma-specific CD40L and IL-2 transgene expression

**Figure 1:**
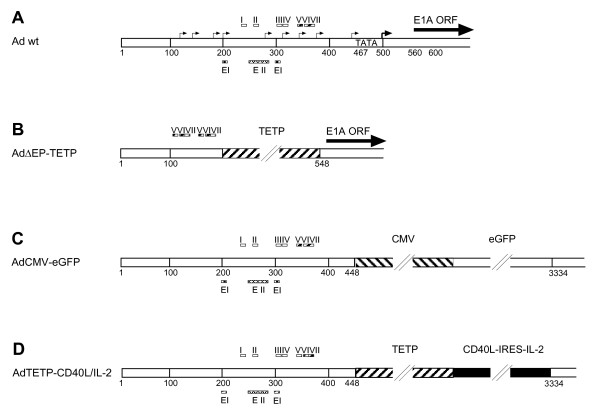
**Structures of E1A promoter of wt Ad5, AdΔEP-TETP and E1 region of AdCMV-eGFP, AdTETP-CD40L/IL-2**. (A) The first 650 bp of the wt Ad genome comprising the left ITR, enhancer elements (EI, EII), packaging elements (I-VII), minor (thin arrows) and major (bold arrow) transcriptional start sites, TATA-box and the beginning of the E1A ORF. (B) Insertion of TETP in combination with duplication of packaging elements V-VII and deletion of endogenous E1A promoter resulting in AdΔEP-TETP. (C) In AdCMV-eGPF, the deleted E1 region from nt 449 to 3333 is replaced with the CMV-eGPF expression cassette. (D) In AdTETP, the deleted E1 region from nt 449 to 3333 is replaced with the TETP-CD40L-IRES-IL-2 expression cassette. The nucleotide numbers refer to the corresponding wt sequence.

**Figure 2:**
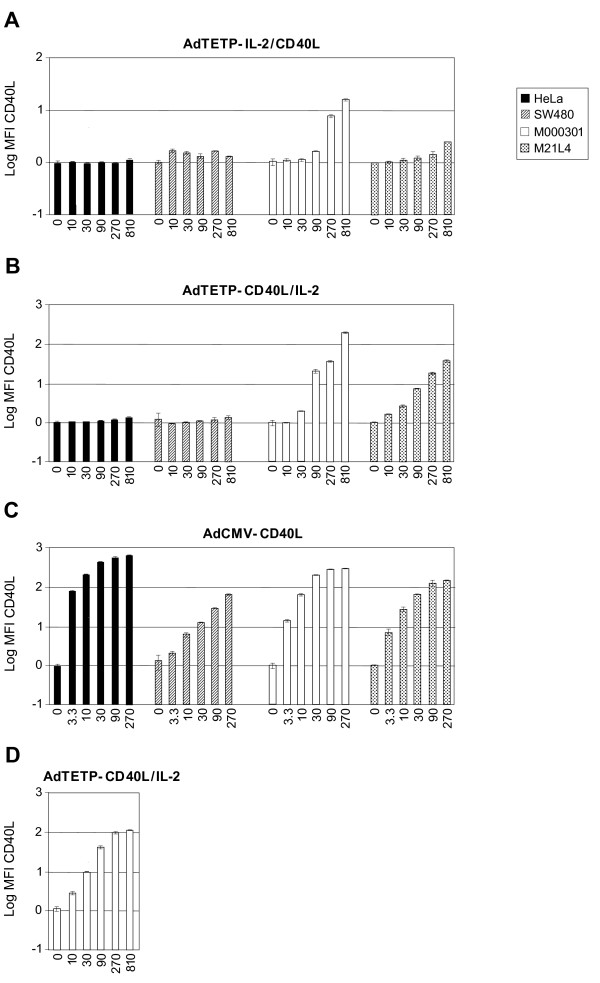
**CD40L transgene expression by different recombinant Ad vectors**. Serial 3-fold increasing amounts of AdTETP-IL-2/CD40L (A), AdCD40L/IL-2 (B), and AdCMV-CD40L (C) were used to transduce HeLa cervical carcinoma, SW480 colon carcinoma, and M000301 and M21L4 melanoma cells for 5 h. Cells were then washed, and CD40L was analyzed two days p.i. by flow cytometry. Results are shown as mean fluorescence intensity (MFI) of triplicate measurements. (D) Transduction of M000301 cells with AdCD40L/IL-2 without washing off virus. Analysis was performed as described for A-C.

Tissue-specific promoters have been utilized to restrict expression of transgenes to target tissues, or to restrict viral replication to targeted cancer cells [45-47]. Here we intended to restrict Ad-mediated expression of immunotherapeutic CD40L and IL-2 genes to melanoma cells by replacing the CMV promoter of a first-generation, E1/E3-deleted Ad5-based vector with the melanoma/melanocyte-specific TETP (Fig. 1) [40]. Two recombinant vectors were generated, AdTETP-IL-2/CD40L and AdTETP-CD40L/IL-2, containing IRES-linked, bicistronic expression cassettes coding for mouse IL-2 and CD40L. Similar IRES-linked expression cassettes have been described in the literature, and it was anticipated that IRES-mediated second gene expression was lower than first gene expression [48]. When non-melanoma HeLa or SW480 cells were transduced with serial 3-fold increasing concentrations of the two novel viruses, no CD40L transgene expression was detected, even at the highest virus input of MOI 810 (Fig 2A, B). However, high levels of CD40L expression were achieved in these cells from control AdCMV-CD40L vector (Fig 2C). Of note, SW480 cells required about 80-fold more AdCMV-CD40L to reach comparable levels as HeLa cells, which are highly Ad sensitive [49]. In contrast to the non-melanoma cells, transduction of melanoma M000301 and M21L4 cells gave rise to robust CD40L expression with both, AdTETP-IL-2/CD40L and AdTETP-CD40L/IL-2 (Fig 2A, B). The expression levels were dependent on the relative CD40L gene position, as CD40L upstream of the IRES sequence gave rise to about 10- and 15-fold higher expression levels in M000301 and M21L4 cells, respectively, than CD40L positioned downstream of IRES. When using AdCMV-CD40L, the strong CMV promoter gave rise to 27- to 40-fold higher expression levels in M000301 and M21L4 cells, respectively, compared to the expression levels achieved with the AdTETP-CD40L/IL-2 vector. The transduction procedure applied here included virus inoculation for 5 h, followed by removal of the virus. In the virus input range from MOI 10 to 270, expression levels of CD40L could be further increased by a factor of 2 to 3 when the inoculum was not removed (Fig. 2B, D), in agreement with a 5 h virus adsorption efficiency of about 50% [50].

**Figure 3:**
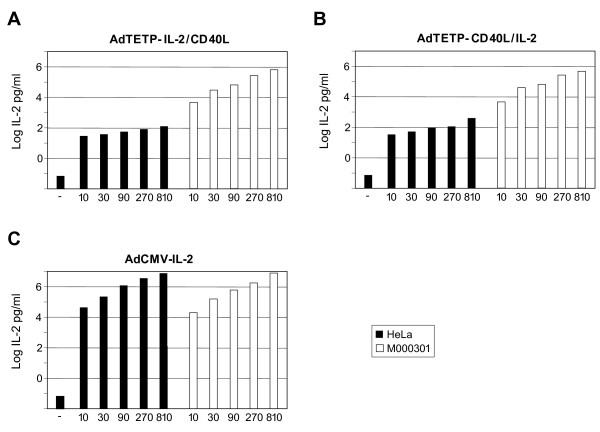
**IL-2 transgene expression by different recombinant Ad vectors**. Serial 3-fold increasing amounts of AdTETP-IL-2/CD40L (A), AdCD40L/IL-2 (B), and AdCMV-IL-2 (C) were used to transduce HeLa cervical carcinoma and M000301 melanoma cells for 5 h. Cells were then washed, and IL-2 of the supernates was analyzed two days p.i. by ELISA. Results are shown as mean of duplicate measurements.

In a next step, IL-2 concentrations contained in the supernates of the above transduced HeLa and M000301 cells were determined. In supernates of HeLa cells, AdTETP-IL-2/CD40L of MOI 10 to 810 gave rise to IL-2 levels from 29 to 144 pg/ml (Fig. 3A). IL-2 production from AdTETP-CD40L/IL-2 was in the range of 36 to 424 pg/ml (Fig. 3B). In supernates of melanoma M000301 cells, both viruses led to very similar expression levels from 5 × 10^3 ^to 7 × 10^5 ^pg/ml (Fig. 3A, B). Thus, for MOIs of 10 and 810, AdTETP-IL-2/CD40L-mediated IL-2 expression levels were 177- and 5652-fold higher in M000301 cells compared to HeLa cells. For AdTETP-CD40L/IL-2, the values were 149- and 1533-fold higher. In contrast to the CD40L gene, no consistent influence of the IL-2 gene position was noticed for IL-2 expression in the two different cell types. Of note also, detection of low IL-2 amounts in supernates of HeLa cells, as opposed to undetectable CD40L expression in these cells, is most likely explained by the difference of sensitivity and/or dynamic range of the assay systems used here. AdCMV-mediated IL-2 expression levels were comparable in the two cell lines tested (Fig. 3C) and reached a minimal 5- to a maximal 12- fold higher levels in melanoma M000301 when compared to TETP-mediated expression levels in these cells. In HeLa cells, AdCMV-mediated IL-2 expression levels were between 1544- and 53472-fold higher compared to TETP-mediated expression levels.

In summary, replacement of the ubiquitously active and strong CMV enhancer promoter with the melanoma/melanocyte-specific enhancer promoter TETP resulted in 5 to- 40-fold lower, but cell type-specific transgene expression.

### Enhancement of GFP transgene expression by adenovirus trans-complementation

**Figure 4:**
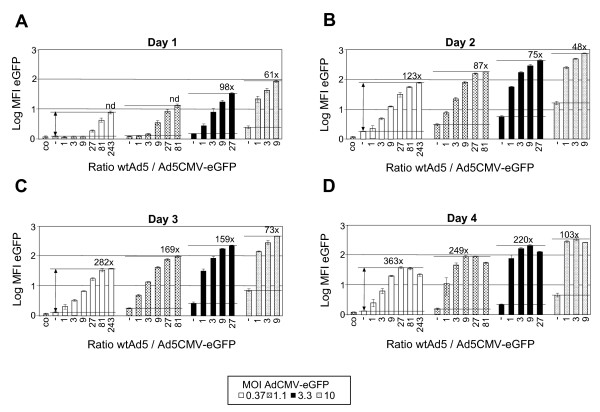
**Concentration-dependence and kinetics of Ad co-replication-mediated enhancement of eGFP expression**. SW480 cells were infected with AdCMV-eGFP at MOIs of 0.37, 1.1, 3.3 and 10 alone (-), or in combination with wtAd5 at concentrations ranging from MOI 0.37 to 90, using serial 3-fold increases. The resulting ratios of wtAd5/AdCMV-eGFP are indicated. eGFP expression analyses were performed at day 1, 2, 3 and 4 p.i. by flow cytometry, and enhancement factors were calculated, following subtraction of auto-fluorescence of uninfected cells (cells only, co), unless, differences between uninfected and infected cells at MOI 0.37 were statistically not significant (not determined, nd).

To determine concentration-dependence and kinetics of Ad trans-complementation-mediated enhancement of gene expression, we first used eGFP as a reporter system. Initial experiments with each four non-melanoma and melanoma cell lines revealed strongest co-replication-mediated enhancement effects in SW480 colon carcinoma cells (not shown). In a next step, we used SW480 cells to determine the kinetics of the enhancement effect in more detail. For this, SW480 cells were infected with AdCMV-eGFP at MOIs of 0.37, 1.1, 3.3 and 10 alone, or in combination with wtAd5 at concentrations ranging from MOI 0.37 to 90, using serial 3-fold increases (Fig. 4). The resulting ratios of wtAd5/AdCMV-eGFP amounted to 1, 3, 9, 27, 81 and 243 for the first set of six samples using AdCMV-eGFP at an MOI of 0.37. Reporter eGFP expression analyses were performed at day 1, 2, 3 and 4, and enhancement was calculated after subtraction of auto-fluorescence of uninfected cells (Fig. 4A-D). WtAd5-mediated enhancement of GFP expression was seen at all four time points, with highest absolute expression levels at day 2, when AdCMV-eGFP at MOIs of 0.37, 1.1, 3.3 and 10 alone resulted in eGFP expression levels of 1.8, 3.1, 5.6 and 15.9 arbitrary mean fluorescence intensity units, respectively. As a result of wtAd5 addition, these expression levels were enhanced by 123-, 87-, 75-, and 48-fold, from lowest AdCMV-eGFP input level of MOI 0.37 to highest AdCMV-eGFP input level of MOI 10. For all four time points, the enhancement effects were strongest for the lowest AdCMVe-GFP input of MOI 0.37, and then gradually decreased with higher input of AdCMVe-GFP. On the other hand, strongest enhancement correlated with the highest ratio of wtAd5/AdCMV-eGFP used, except at day 4, where enhancement effects at the highest ratios started to decline due to cytopathic effects induced by the high wtAd5 input of MOI 90. When using a ratio of 1:1 for reporter vector to wtAd5, highest enhancement found was at day 4 amounting to 84-fold at MOI 10 of each virus.

**Figure 5:**
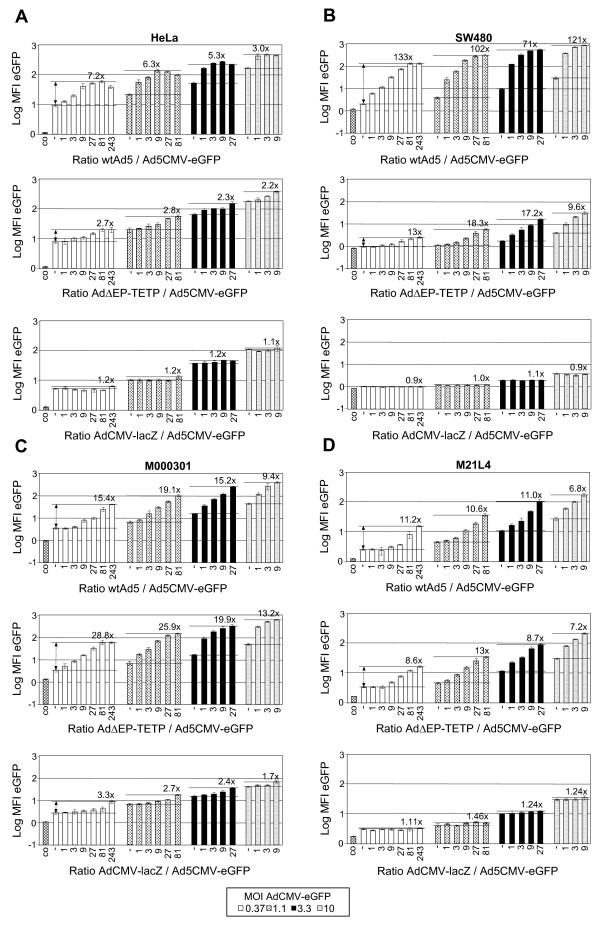
**Comparison of co-replication enhancement by wtAd5 and melanoma RC Ad virus AdΔEP-TETP**. The indicated non-melanoma and melanoma cells were co-infected using AdCMV-eGFP at MOIs of 0.37, 1.1, 3.3 and 10 in combination with RC wtAd5 and AdΔEP-TETP, or the RD E1-deleted AdCMV-lacZ. Concentrations of the latter viruses were in the range from MOI 0.37 to 90, using serial 3-fold increases and resulting virus ratios as in Fig. 4. eGFP expression was recorded at day 2 by flow cytometry, and enhancement factors were calculated as in Fig. 4.

We then tested the performance of our previously described melanoma replication-competent (RC) Ad virus AdΔEP-TETP [40] in trans-complementation-mediated expression assays. For co-infection, AdCMV-eGFP at MOIs of 0.37, 1.1, 3.3 and 10 were combined with RC wtAd5 and AdΔEP-TETP, or as control, the replication-defective E1-deleted AdCMV-lacZ at concentrations ranging from MOI 0.37 to 90, using serial 3-fold increases as in the previous experiment. Reporter eGFP expression was recorded at day 2. The tested cells included HeLa cervical carcinoma cells, SW480 and DLD-1 colon carcinoma cells, and A549 lung carcinoma cells, and four melanoma cells M000301, MeWo, M21L4 and SK-Mel23 (Fig. 5, Table 2).

**Table 2:** Highest trans-complementation-mediated enhancement of GFP expression of day 2 experiment

	Virus	WtAd5			AdΔEP-TETP			AdCMV-lacZ		
**Cell line**	**MFI expression AdCMV-eGFP MOI 0.37**	**Highest fold enhancement**	**Input MOI AdCMV- eGFP of highest fold enhancement**	**Ratio WtAd5/AdCMV-eGFP**	**Highest fold enhancement**	**Input MOI AdCMV- eGFP of highest fold enhancement**	**Ratio** **AdΔEP-TETP/AdCMV-eGFP**	**Highest fold enhancement**	**Input MOI AdCMV- eGFP of highest fold enhancement**	**Ratio** **AdCMV-lacZ/AdCMV-eGFP**

HeLa (cervical carcinoma)	9.0	7.2	0.37	81	2.8	1.1	81	1.2	1.1/3.3	81/9

SW480 (colon carcinoma)	2.9	133	0.37	243	18.3	1.1	81	1.1	3.3	9

A549 (lung carcinoma)	7.4	25.1	3.3	3	6.9	1.1	81	nd		

DLD-1 (colon carcinoma)	15	7.7	3.3	9	3.8	0.37	243	nd		

M000301 (melanoma)	3.3	19.1	1.1	81	28.8	0.37	243	3.3	0.37	243

M21L4 (melanoma)	3.3	11.2	0.37	243	13	1.1	81	1.5	1.1	27

SK-Mel23 (melanoma)	1.62	6.4	3.3	27	15.4	1.1	27	nd		

MeWo (melanoma)	54	3.1	0.37	243	3.5	1.1	27	nd		

Nd: not determined

WtAd5 enhanced expression in all eight cell lines, although at variable extents depending on the ratios of RC virus/reporter vector. Enhancement factors were in the range of 133-fold in SW480 to 3.1-fold in MeWo. Cells with enhancement factors < 10 included HeLa, DLD-1, SK-Mel23 and MeWo cells, which all, except SK-Mel23, revealed relatively high transduction sensitivity to the lowest dose of MOI 0.37 AdCMV-eGFP virus input, resulting in 9, 15 and 54 MFI units, respectively (Table 2). Cells with enhancement factors >10 included SW480, A549, M000301 and M21L4 and were less transduction-sensitive at this virus dosage, giving rise to 2.9, 7.4, 3.3, and 3.3 MFI units, respectively. In melanoma cells, enhancement mediated by AdΔEP-TETP was similar, and in three of them even increased, when compared to wtAd5. In non-melanoma cells, enhancement factors with this virus were about 1.5- to 10-fold lower, always with the highest RC/reporter vector ratio used. This residual enhancement effect mediated by the AdΔEP-TETP CRAd is most likely due to low E1A expression from this vector, as replacement of the RC virus with the RD AdCMV-lacZ led to only very minor enhancement effects on transgene expression. This is in agreement with findings that traces of E1A expression are sufficient to induce replication of the therapeutic vector genome [40,51]. As seen for SW480 cells, enhancement was again in general strongest at the highest ratio of RC/reporter vector used, unless the RC virus induced cytopahtic effects.

In summary, robust trans-complementation-mediated reporter expression enhancement was observed using the CMV-eGFP expression cassette, which, however, varied considerably depending on cell types and virus dosages. For melanoma cells, our previously described melanoma-specific AdΔEP-TETP revealed trans-complementation enhancement comparable to wtAd5.

### Comparison of adenovirus trans-complementation-mediated enhancement of CD40L and IL-2 from CMV and TETP promoters

**Figure 6:**
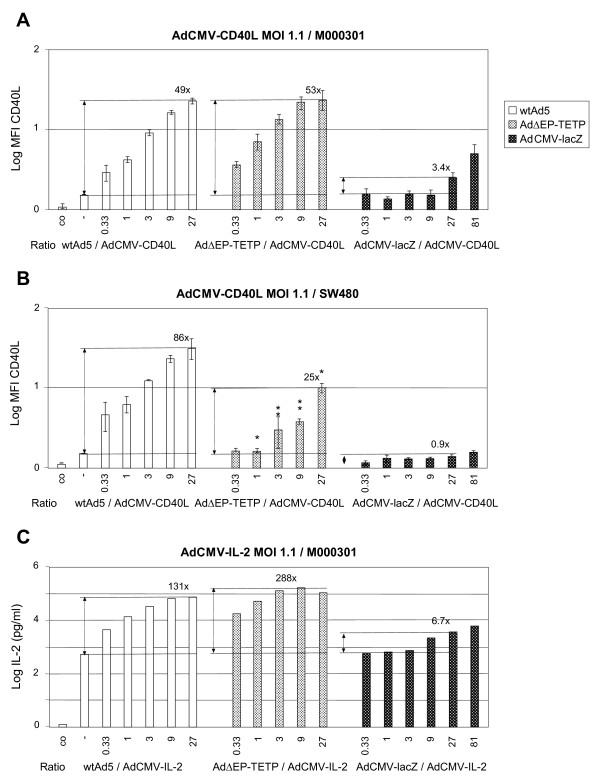
**Adenovirus co-replication enhancement of immune modulators CD40L and IL-2 expressed from CMV promoter cassette**. Melanoma M000301 or colon SW480 cells were infected with AdCMV-CD40L (A, B), or AdCMV-IL-2 (C), at an MOI of 1.1, and were combined with 3-fold increasing amounts of RC wtAd5 and AdΔEP-TETP, or the replication-defective E1-deleted AdCMV-lacZ. The resulting ratios of RC/transgene vector are indicated. CD40L and IL-2 transgene expression analyses were performed two days p.i. by flow cytometry and ELISA, respectively. Asterisks indicate the level of significance (*P < 0.05; **P < 0.005; for comparison with corresponding wtAd5/AdCMV-CD40L values).

Next we tested whether Ad trans-complementation also enhanced the expression of the immune modulator genes CD40L and IL-2. In a first experiment, AdCMV-CD40L (Fig. 6A, B) or AdCMV-IL-2 (Fig. 6C) at MOI 1.1 were combined with RC wtAd5 and AdΔEP-TETP, or the RD E1-deleted AdCMV-lacZ at concentrations ranging from MOI 0.37 to 90 using serial 3-fold increases. The resulting ratios of RC/transgene vector were in the range from 0.33 to 27 when including RC viruses, and 0.33 to 81 when including RD AdCMV-lacZ control virus. At this low input of CD40L and IL-2 transgene vectors, trans-complementation resulted in high enhancement of immunostimulatory gene expression in both cell lines. Trans-complementation-mediated maximal expression enhancement for CD40L was 86-fold in SW480 cells, and 53-fold in M000301 cells. For IL-2, maximal enhancement amounted to 288-fold in M000301 cells. For non-melanoma SW480 cells, a significantly smaller CD40L expression enhancement was found when replacing wtAd5 with AdΔEP-TETP. Inclusion of AdCMV-lacZ as control revealed only very minor enhancement effects, reaching in M000301 cells about 7 and 5% of wtAd5-mediated enhancement of CD40L and IL-2 expression, respectively (Fig. 6A, C).

**Figure 7:**
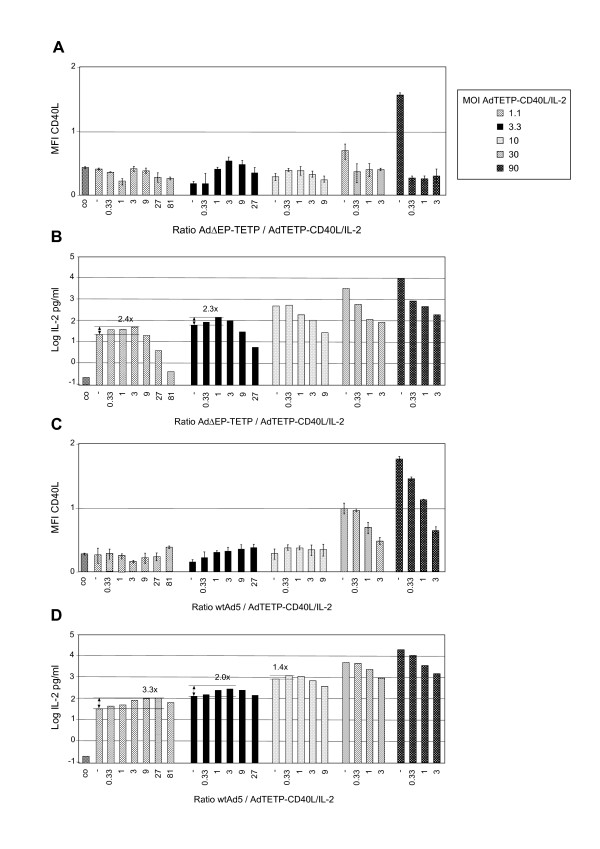
**Adenovirus co-replication enhancement of immune modulators CD40L and IL-2 expressed from the tissue-specific TETP promoter cassette**. Melanoma M000301 cells were infected with AdTETP-CD40L/IL-2 at the indicated MOIs and were combined with 3-fold increasing amounts of RC AdΔEP-TETP (A, B), or wtAd5 (C, D). The resulting ratios of RC/transgene vector are indicated. CD40L and IL-2 transgene expression analyses were performed two days p.i.. This experiment was performed twice, with similar results.

To see whether Ad trans-complementation could also enhance expression from a vector containing a tissue-specific promoter, the experiment was repeated with the transgene vector AdTETP-CD40L/IL-2. In contrast to the previous experiments, low but consistent enhancement effects were found for the expression of IL-2, and none for CD40L (Fig. 7A, B). When AdTETP-CD40L/IL-2 was combined with AdΔEP-TETP in M000301 cells, trans-complementation-mediated enhancement of IL-2 amounted to 2.4- and 2.3-fold for the two lowest AdTETP-CD40L/IL-2 virus concentrations of 1.1 and 3.3, respectively. To exclude that the low enhancement effect is a peculiar feature restricted to M000301 cells, trans-complementation assays were also conducted in SK-Mel23 and M21L4 cells, with similar results (data not shown).

**Figure 8:**
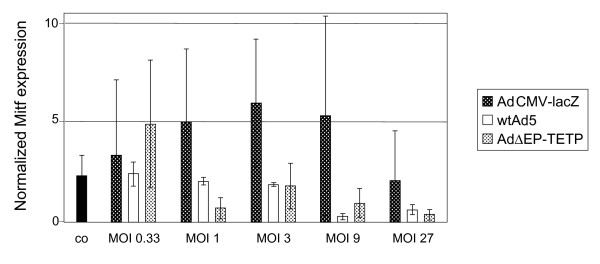
**Mitf RNA expression levels in non-infected (co) and infected cells as measured by RT-PCR**. Cells were harvested two days p.i..

In order to evaluate possible competitions for limited transcription factors controlling TETP, the experiment was repeated with wtAd5 replacing AdΔEP-TETP. Again, no enhancement was seen for CD40L expression, and low 3.3-, 2.0- and 1.4-fold enhancement factors were recorded for IL-2 levels at virus inputs of 1.1, 3.3 and 10, respectively (Fig. 7C, D). IL-2 expression levels decreased for all samples with AdΔEP-TETP ≥ 10, due to cytopathic effects, whereas for wtAd5, cytopathic effects appeared in general at higher concentrations of ≥ 30 and were less strong. These findings do not a priori exclude that competition for an essential transcription factor is responsible for the low enhancement effect, in case such a factor is simultaneously involved in controlling TETP as well as viral promoters (see discussion). However, it has earlier been reported that ectopic expression of Ad E1A resulted in down regulation of Mitf, which binds to the tyrosinase promoter and is a member of the basic helix-loop-helix transcription factor family [52]. We thus tested Mitf levels in infected M000301 cells by real-time RT-PCR (Fig. 8). Although not of statistical significance, there was a clear trend for lower levels of Mitf expression in M000301 cells infected with the higher concentrations of the E1A-expressing viruses wtAd5 and AdΔEP-TETP, compared to E1A-deleted AdCM-lacZ or uninfected cells. As Mitf expression levels strongly influence activity of the tyrosinase promoter [53], this could contribute to the lack of strong trans-complemenation enhancement in the TETP trans-complementation system described here.

## Discussion

Strategies to improve experimental cancer therapy include co-delivery of RC oncolytic Ads together with RD vectors expressing therapeutic genes [17,21,22,26,28,30,31,33-39]. There is a large choice of candidate therapeutic genes that has been evaluated, including tumor-suppressor, immunomodulatory or cytotoxic genes. Depending on the type of cytotoxic genes used, premature death of transduced cells was found to reduce replication of oncolytic viruses, thereby antagonizing the therapeutic efficacy [54]. Genes with indirect antitumor effects such as immune stimulators might thus be advantageous. We have shown earlier that the combination of IL-2 and CD40L had an improved efficacy over the use of single agents, when applied for direct *in situ *therapy or vaccination therapy in a mouse melanoma model [44]. Moreover, we showed that intratumoral injection of an IL-2-expressing Ad vector could induce tumor regression in patients with advanced melanoma [3]. The currently 287 ongoing or closed gene therapy protocols utilizing Ad vectors for cancer treatment include 20 protocols with IL-2, and 11 protocols with CD40L as therapeutic gene, respectively. Five protocols, mainly for treatment of leukemia, include combined expression of IL-2 and CD40L http://www.wiley.co.uk/genmed/clinical/.

Utilization of a binary vector system as compared to direct therapeutic expression from single oncolytic viruses poses disadvantages, including more demanding vector manufacturing and clinical handling. On the other side, possible advantages compared to armed oncolytic vectors include their flexible application in combination with an increase of the overall therapeutic gene capacity of the delivery system. For example, a single CRAd can be combined with numerous therapeutic RD vectors, which have been tested previously as single agents. Alternatively, efficacy of different CRAds could be compared side-by-side in combination with the same therapeutic vector. The binary vectors may also be safer than single viruses, as the amount of vector expressing the therapeutic gene can be delayed to later rounds of virus application, and can, in addition, be dose-adjusted. Dissemination of the therapeutic vector, however, remains a risk, in particular if strong ubiquitous promoters are used to control expression. Thorne et al., e.g., found that co-injection of an oncolytic AdΔ24 together with RD CMV promoter/enhancer-controlled luciferase vector into xenotransplanted tumors not only gave rise to enhanced and more durable gene expression in the tumor tissue, but also led to secondary, weaker bioluminescence in the liver [26]. Thus, additional safety measures are warranted for this kind of approach.

Here we suggest to utilize our previously described melanoma/melanocyte-specific TETP [40] to control expression of CD40L/IL-2 from a bicistronic expression vector. About 30 tissue-specific mammalian promoters have been evaluated in RC CRAds [47], and many more were tested for their fidelity to express transgenes from RD vectors (reviewed in [46]). The quest for such promoters was brought up following the findings that *in vivo*, expression from vectors containing the ubiquitously active and strong CMV promoter resulted in efficient transduction that peaked within a few days, but frequently became undetectable by one month after injection. These findings were ascribed to either elimination of viral genomes by the immune system or to methylation-induced promoter silencing, and it was suggested that this could be overcome by using tissue-specific mammalian promoters [55,56].

We found that replacement of the CMV enhancer promoter with the TETP allowed tight melanoma-specific transgene expression, as IL-2 expression levels amounted to a minimal 149-fold to a maximal 5652-fold higher expression levels in melanoma cells compared to HeLa cells. This is comparable with earlier reports, where a recombinant Ad expressing β-Gal under the control of two copies of the mouse tyrosinase enhancer in combination with a 770 bp mouse tyrosinase promoter gave rise to 100- to 200-fold higher expression in melanoma cells compared to non-melanoma cells [57]. In our experiments, a comparison of TETP-controlled transgene expression levels with those derived from CMV-controlled vectors revealed, depending on cell line and transgene, 5 to- 40-fold lower expression levels in melanoma cells. This is in line with findings that tissue-specific promoters frequently turned out to be relatively weak promoters compared to CMV or other strong viral promoters [58]. A slightly different version of tyrosinase promoter/enhancer combination has been claimed to reach, at least in a very limited number of melanoma cells, comparable expression levels as from CMV promoter [57], whereas others found only maximal 1.3% of the CMV promoter-driven transcriptional activity [59]. In our hands, TETP-controlled luciferase expression was found to give rise to about 10- to 20-fold higher expression levels compared to SV40 promoter [40].

Variations in the expression levels of IRES upstream and downstream genes have been observed previously. In one study, IRES-dependent second gene expression has been reported to range from 6 to 100% of the first gene expression. This was found to be influenced by several factors, including the choice of optimal/nonoptimal Kozak sequence of the ATG start codon, as well as the particular cell type used for expression [48]. In contrast, in IRES-encoding RNA viruses, yields of translation product from the downstream IRES-dependent cistron were also found to be higher than from the upstream cistron [60].

When we used the AdCMV-eGFP/wtAd5 binary vector system, robust trans-complementation-mediated reporter expression enhancement was obtained in melanoma and non-melanoma cells. In our eight cell types tested, four revealed enhancements in the range of 3 to 10, and four had enhancements larger than 10, with a maximal 363-fold for SW480 cells on day 4. Such a strong variation of trans-complementation-mediated enhancement has been found in other studies, and may depend on cell type used, exact transgene vector: CRAd ratio, time point of individual virus additions (variable in some studies), trans-activation of the CMV promoter by cellular and viral gene products [51], and most importantly, also the type and dynamic range of analysis system utilized for quantification. Enhancement values of > 50, e.g., were obtained by others when using luciferase assays [22,24,28], ELISA [38,39], and virus progeny titers [19] which have the largest dynamic range.

When replacing the transgene vector AdCMV-eGFP with AdCMV-CD40L or AdCMV-IL-2, 49- and 131-fold trans-complementation-mediated transgene expression enhancement was found in melanoma M000301 cells, when combined with wtAd5, and 53- and 288-fold enhancement when combined with AdΔEP-TETP. To our surprise, combination of the newly established AdTETP-CD40L/IL-2 with AdΔEP-TETP or wtAd5 revealed an unexpectedly low trans-complementation-mediated expression enhancement of maximal 3.3 fold for the more sensitive IL-2 expression analysis. Possible reasons for this low enhancement effect could include competition for virus binding, similar entry pathways or post entry factors such as transcription factors or nuclear domains for replication. We consider entry competition unlikely, since trans-complementation with CMV-promoters from Ad5 capsids was highly efficient. All viruses tested here utilize the Coxsackie virus B Ad receptor as attachment receptor and αv integrins as entry receptors [61].

The unique difference between the AdCMV-eGFP and AdTETP-CD40L/IL-2 vectors relates to the sequences of the CMV-eGFP and TETP-CD40L/IL-2 expression cassettes, whereas the rest of the viral genome is identical (Fig. 1). Transcription factors could be a limiting factor if they bound in a rate-limiting manner to the TETP sequence (but not to the CMV enhancer/promoter sequence), and at the same time also to one of the viral promoters. The human tyrosinase promoter sequence consists of the M-box (a conserved element found in other melanocyte-specific promoters) containing a first E-box motif, an SP1 site, and a highly conserved CR2 element comprising a second E-box motif and an overlapping octamer element [62]. Both E-boxes were demonstrated to bind the basic helix-loop-helix transcription factor Mitf, which is essential for melanocyte differentiation. Of note, a related E-box motif is also contained in the Ad major late promoter (MLP), which usually is bound by USF, another ubiquitous member of the basic helix-loop-helix transcription factor family [63]. Based on band shift assays, it was found that Mitf also could bind to the E-box of the MLP, and conversely, the USF also could bind to the M-box of melanocyte-specific promoters [53]. Similarly, band shift assays with an oligonucleotide containing the SP1 motif of the tyrosinase promoter were competed by a SP1 motif found in the Ad EII late promoter [53]. The tyrosinase enhancer sequence is less clearly characterized, but was suggested to contain a binding site for a member of the fos family transcription factor [64]. The strong CMV enhancer/promoter contains multiple transcription factor binding sites, including the ubiquitous Sp1 family of transcription factors, NF-κB, retinoic acid nuclear receptors and CREB/ATF [65]. Thus, depending on the cell type and expression levels of members of the basic helix-loop-helix transcription factor or SP1 family, competition for such factor(s) could possibly contribute to the lower enhancement of the Ad trans-complementation system found here.

However, a more likely explanation relates to the findings by the group of Goding, which reported that ectopic expression of Ad E1A resulted in down regulation of Mitf in mouse melan-a melanoma cells, leading to repression of TRP-1 and tyrosinase levels and subsequent loss of pigmentation [52]. This repression could be relieved by over expression of Mitf. We confirmed down regulation of Mitf transcripts in human M000301 melanoma cells following infection with E1A-expressing wtAd5 and AdΔEP-TETP, but not with E1A-deleted AdCMV-lacZ. Whether this is the exclusive mechanism to explain lack of strong enhancement, or whether there is an additive effect together with the above discussed competition for transcription factors remains to be further studied. In addition, as strong and specific delivery of therapeutic genes is one of the main goals of cancer therapy, it may be of importance for the general usage of the binary Ad expression system to investigate whether this finding is unique to the TETP system tested here, or whether it also occurs with other tissue- or cell type-specific promoters.

## Conclusions

In the current study we were able to demonstrate strong *in vitro *enhancement of eGFP, IL-2 and CD40L transgene expression by the Ad trans-complementation system when using CMV-controlled expression cassettes in combination with RC Ads. When using TETP as cancer cell-selective promoter to restrict IL-2 and CD40L transgene expression to melanoma cells, tight expression was demonstrated, which was only 5 to 40-fold lower than those from vectors controlled by the nonselective CMV promoter. However, when combining the TETP-controlled transgene vectors with RC wtAd5 or the AdΔEP-TETP CRAd vector, very moderate trans-complementation enhancement was noticed in melanoma cells. At least in part, this phenomenon may be due to Ad-mediated down-regulation of the limiting transcription factor Mitf. It remains to be investigated, whether this finding is unique for the particular promoter system used here, or whether it also occurs with other promoter systems. Tight cancer cell-selective promoter control remains a highly desirable feature to restrict transgene expression to the targeted tumor tissue and avoid collateral damage.

## Abbreviations

Ad: adenovirus; CMV: cytomegalovirus; CRAD: conditionally replicating Ad; CD40L: CD40 ligand; GM-CSF: granulocyte macrophage colony stimulating factor; IL-2: interleukin-2; IRES: internal ribosomal entry site; MFI: mean fluorescence intensity; MITF: Microphthalmia-associated bHLH-LZ transcription factor; MLP: major late promoter; MOI: multiplicity of infection; P.I.: post infection; pfu: plaque forming unit; RC: replication-competent; RD: replication-deficient; TETP: tyrosinase enhancer tyrosinase promoter;

## Competing interests

The authors declare that they have no competing interests.

## Authors' contributions

ACF and SH designed the experiments, generated the Ad vectors and carried out the transduction studies. VL performed the IL-2 ELISA, and OS carried out the RT-PCR measurements. SH coordinated the study and wrote the manuscript. RD and UFG participated in the design and coordination and helped to draft the manuscript. All authors read and approved the final manuscript.
